# How Age Affects Personal and Social Reactions to COVID-19: Results from the National Understanding America Survey

**DOI:** 10.1093/geroni/igaa057.3470

**Published:** 2020-12-16

**Authors:** Jung Ki Kim, Eileen Crimmins

**Affiliations:** 1 University of Southern California, Los Angeles, United States; 2 University of Southern California, Los Angeles, California, United States

## Abstract

The pandemic of COVID-19 has had tremendous impact on Americans’ lives including their personal and social behaviors. While everyone is affected in some way by the pandemic, older persons have been far more likely to suffer the most severe health consequences. For this reason, how people have responded to the COVID-19 outbreak may differ by age. Using a nationally representative sample from the Understanding America Study (UAS), we examined differentials in behavioral responses to COVID-19 by age and how they change over time. At the beginning of the pandemic (March, 2020), older people were less likely than younger ones to engage in preventive behaviors. As the pandemic progressed, however, older people have adopted healthy behavioral changes more than younger people, such that about two months after the pandemic started, older people were more likely to comply with suggested and regulated behaviors including practicing better hygiene, quarantining, and social distancing. Even when considering other potential influences on behavioral responses, older age was significantly related to performing more preventive behaviors, and gender, racial/ethnic minority status, perceived risk for infection and dying and political orientation were also found to be related to people’s behavioral responses.

